# Iliofemoral Tortuosity Increases the Risk of Access-Site-Related Complications After Aortic Valve Implantation and Plug-Based Access-Site Closure

**DOI:** 10.1016/j.cjco.2022.03.006

**Published:** 2022-03-19

**Authors:** Arpad Lux, Lisa Müllenberg, Leo F. Veenstra, Wim Dohmen, Suzanne Kats, Bart Maesen, Arnoud W.J. van’t Hof

**Affiliations:** a Department of Cardiology, Maastricht University Medical Center+, Maastricht, The Netherlands; b Cardiovascular Research Institute Maastricht University, Maastricht, The Netherlands; c Department of Cardiothoracic Surgery, Maastricht University Medical Center+, Maastricht, The Netherlands; d Business Information Management, Maastricht University Medical Center+, Maastricht, The Netherlands; e Department of Cardiology, Zuyderland Medical Centrum, Heerlen, The Netherlands

## Abstract

**Background:**

Access-site-related complications are often related to high-risk anatomy and technical pitfalls and impair the outcomes of transfemoral aortic valve implantations (TAVIs). Calcification and tortuosity are widely recognized risk factors, and their impact on procedural planning is left to the implanting experts’ discretion. To facilitate decision-making, we introduced a quantitative measure for iliofemoral tortuosity and assessed its predictive value for access-site-related vascular and bleeding complications.

**Methods:**

We performed a single-centre prospective cohort study of consecutive, percutaneous transfemoral TAVI performed between April 2019 and March 2020. Medical history and all-cause mortality were extracted from the electronic patient files. Arterial anatomy and calcifications were evaluated using 3mensio Structural Heart software. The primary outcome was access-site-related vascular or bleeding complications.

**Results:**

In this elderly, intermediate-risk population, we registered the primary outcome in 43 patients (39%), and major access-site complications in 10 patients (9.2%). Complete hemostasis was achieved in 77 patients (70.6%), by the application of the MANTA plug alone. In the group with access-site-related adverse events, compared with the group without, the tortuosity index was higher median (26% interquartile range [IQR 18%-33%] vs median 19% [IQR 13%-29%], respectively; *P* = 0.012), as was maximal angulation median (50° [IQR 40°-59°] vs median 43° [IQR 36°-51°], respectively; *P* = 0.026) were higher. Both variables had a significant effect on our primary outcome, with odds ratios (OR) of 3.1 (tortuosity, *P* = 0.005) and 2.6 (angulation, *P* = 0.020). The degree of angulation was a predictor of major complications too (odds ratio 7 [1.4-34.8]; *P* = 0.017).

**Conclusions:**

Steeper angles and greater arterial elongation increase the risk of vascular and bleeding complications after femoral TAVI with the utilization of a plug-based closure device.

## Résumé

**Introduction:**

Les complications liées au site d’accès qui sont souvent associées à une anatomie qui expose à des risques élevés et aux pièges techniques nuisent aux résultats cliniques des implantations valvulaires aortiques par cathéter (TAVI) par voie fémorale. Il est largement reconnu que la calcification et la tortuosité sont des facteurs de risque. Par conséquent, leurs conséquences sur la planification interventionnelle sont laissées à la discrétion des experts en implantation. Pour faciliter la prise de décision, nous avons mis en place une mesure quantitative de tortuosités iliofémorales et évalué sa valeur prédictive sur la survenue de complications vasculaires et hémorragiques liées au site d’accès.

**Méthodes:**

Nous avons réalisé une étude de cohorte prospective unicentrique de TAVI percutanées par voie fémorale consécutives effectuées entre avril 2019 et mars 2020. Nous avons extrait les antécédents médicaux et la mortalité toutes causes confondues des dossiers médicaux électroniques. Nous avons évalué l’anatomie artérielle et les calcifications à l’aide du logiciel 3mensio Structural Heart. Le critère de jugement principal était les complications vasculaires ou hémorragiques liées au site d’accès.

**Résultats:**

Dans cette population âgée exposée à un risque intermédiaire, nous avons enregistré le critère de jugement principal de 43 patients (39 %), et les principales complications du site d’accès de 10 patients (9,2 %). Soixante-dix-sept patients (70,6 %) ont obtenu l’hémostase complète par la seule application du bouchon MANTA. L’indice de tortuosité était plus élevé dans le groupe qui avait subi des événements défavorables liés au site d’accès que dans le groupe qui n’avait pas subi d’événements défavorables (26 % [18 %-33 %] *vs* 19 % [13 %-29 %], respectivement; *P* = 0,012). Il en était de même pour l’angulation maximale (50° [40°-59°] *vs* 43° [36°-51°], respectivement; *P* = 0,026) qui était plus grande. Les deux variables ont eu des effets significatifs sur le critère de jugement principal, soit des rapports de cotes (RC) de 3,1 (tortuosité, *P* = 0,005) et de 2,6 (angulation, *P* = 0,020). Le degré d’angulation était aussi un prédicteur de complications majeures (rapport de cotes 7 [1,4-34,8]; *P* = 0,017).

**Conclusions:**

Des angles plus prononcés et une plus grande élongation des artères augmentent le risque de complications vasculaires et hémorragiques après la TAVI par voie fémorale lors de l’utilisation d’un dispositif de fermeture à bouchon.

Transcatheter aortic valve implantation (TAVI) is a well-accepted, truly minimally invasive method to treat severe symptomatic aortic valve stenosis.^1^, ^2^, ^3^ The implantation can be performed via multiple access sites, but the transfemoral (TF) approach is not only the most common but also the safest option.^4^ Despite its relative safety, access-site-related vascular and bleeding complications have an unfavourable effect on the outcomes of TF TAVIs, and a large number of patients could benefit from the prevention of these complications.^5^, ^6^, ^7^ We know that patient-related factors and technical pitfalls, usually in combination with the specific properties of the applied closure device or technique, increase the risk of these complications.^8^, ^9^, ^10^, ^11^, ^12^ Interestingly, the role of iliofemoral tortuosity and the amount of vascular calcification remained, until this year, unexplored.^10^, ^11^, ^12^, ^13^ Therefore, we designed a study to prove that vascular calcifications and arterial tortuosity are relevant predictors of vascular and bleeding complications following TF TAVI and plug-based access-site closure.

## Methods

### Study design and population

We performed a single-centre prospective cohort study of consecutive, percutaneous TF TAVIs performed between April 2019 and March 2020. The only inclusion criterion was that access-site closure had to be attempted with a plug-based device (MANTA vascular closure device, Teleflex Inc., Wayne, PA). Relevant medical history and all-cause mortality data were extracted from the electronic patient files. The study was approved by the ethical committee of Maastricht University and Maastricht UMC+; approval number: METC 2019-1253.

### Valve implantation and access-site handling

In a dedicated hybrid operating theatre, all TF valve implantations were performed with patients under conscious sedation. At least one of the operators was highly experienced (> 3 years of experience and > 40 TF implantations per year; 4 members of the team). Echo-guided arterial puncture was left to the operator’s discretion. Patients were treated with either a balloon-expandable (Edwards Lifesciences, Irvine, CA) or a self-expandable (Medtronic plc, Minneapolis, MN) valve. Per protocol, valves were advanced via their manufacturer-dedicated sheaths (e-sheath [Edwards Lifesciences] or InLine sheath [Medtronic] / sheathless). If the usual preoperative assessment suggested significant tortuosity, then the self-expandable valves were advanced via a guiding-sheath from Cook Medical (Bloomington, IN). Per protocol, a plug-based vascular closure device was used (MANTA). Device deployment depth was based on the stop-flow measurement. Until December 2019, 2.0 cm was added to the measured depth; after December 2019, this depth was reduced to 1 cm per a change in the manufacturer instructions.^14^ Any needed venous puncture was performed on the contralateral side. Anticoagulation and any other antiplatelet therapy other than acetylsalicylic acid were discontinued before the procedure. Anticoagulation was bridged in the following specific situations: atrial fibrillation with a **C**ongestive Heart Failure, **H**ypertension, **A**ge (≥ 75 Years) (doubled), **D**iabetes Mellitus, **S**troke (doubled), **V**ascular Disease, **A**ge (65-74) Years, **S**ex **C**ategory (Female) (CHA_2_DS_2_-VASc) score ≥ 8; mechanical heart valves (not in aortic position); and venous thromboembolism for ≤ 3 months. Bridging was carried out using low-molecular-weight heparin until the day of the procedure. Compression bandage was not routinely placed.

### Assessment of vascular and bleeding complications

Arterial closure was scored with a semi-subjective classification: complete arterial hemostasis (minimal subcutaneous oozing permitted); manual compression for > 10 minutes; and pressure bandage or surgical intervention required. Vascular and bleeding complications of the TAVI access site were closely monitored and registered until the fourth postoperative day. Complications of the adjunctive arterial access sites were not included in this analysis, including daily inspection and palpation of the inguinal area and monitoring of hemoglobin levels. An ultrasound examination was done in cases of suspicion of a possible pseudoaneurysm. Pseudoaneurysms were treated according to the advice of vascular surgeons (eg, thrombin injections or surgical repair). At 6 weeks of regular follow-up, patients were asked if they had any access-site-related problems, which were then examined and/or treated by a medical professional.

### Assessment of iliofemoral anatomy

**Figure 1:**
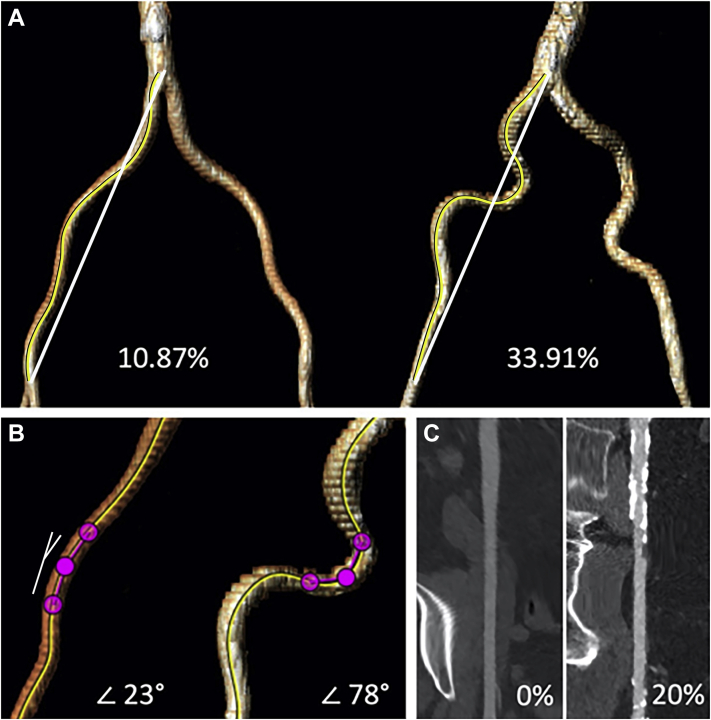
Vascular assessment of the preoperative multidetector computed tomography angiography. (**A**) Tortuosity index: ([centreline {shown by the **yellow line**} of flow distance / direct distance {shown by the **white line**}] – 1) ∗100; an example of a patient with (**left**) a low tortuosity index and (**right**) a high tortuosity index. (**B**) Maximal angulation; measured over the centreline with an arm length of 15 mm; an example of a patient with (**left**) a low maximal angulation and (**right**) a high maximal angulation. The measured angle is demonstrated on the **left side** (**white lines**). (**C**) Calcification index; calcium volume and total vessel volume were measured using the Hounsfield unit; an example of a patient with (**left**) a non-calcified iliofemoral tract and (**right**) a severely calcified iliofemoral tract.

All patients who were accepted for TAVI were screened with multidetector computed tomography angiography to assess the access routes and determine appropriate valve sizes. These images were analyzed using 3mensio Structural Heart software (version 10.0, Pie Medical Imaging BV, Maastricht, The Netherlands). Tortuosity index, angulation, and calcification index were determined for the iliofemoral tract of the access site. We adapted 2 quantification methods to adequately measure tortuosity and calcification. To acquire a tortuosity index, the true arterial length (centreline of flow) and the direct distance between the aortic bifurcation and the femoral bifurcation were measured, and their relation was determined: ([centreline of flow / direct distance] - 1) ∗ 100).^15^^,^^16^ Maximal angulation was measured over the centreline with an arm length of 15 mm. (Fig. 1)

Calcification was measured between the aortic bifurcation and the femoral bifurcation, as this trajectory corresponds well to the length of sheath used for the introduction of the delivery device, and both locations are easily and reproducibly identified. Calcium volume (in mm^3^) was quantified using an individual Hounsfield unit (HU) threshold to compensate for the contrast-agent density. The HU of 3 noncalcified sections in the selected area was measured and averaged, and an additional 200 HU was added to this number, and a volume filter for lesions smaller than 5 mm^3^ was applied.^17^ To obtain the calcification index, the calcium volume was divided by the total volume of the selected area (total vessel volume) and multiplied by 100. We also performed a semiquantitative calcification scoring as previously described: 0, no calcification; 1, mild calcification; 2, moderate calcification; and 3, severe calcification.^8^^,^^18^ Finally, the minimal arterial lumen diameter of the iliofemoral tract was measured.

### Endpoints

The primary outcome was any access-site-related vascular or bleeding complication, as defined by the most recent Valve Academic Research Consortium (VARC)-2 criteria.^19^ For each patient, the most severe periprocedural complication was scored. Secondary outcomes included the state of the access site directly after MANTA closure, post-procedural hospitalization. Complications related to the non-TAVI access sites were not included in the analysis.

### Statistical analysis

Statistical analysis was performed using SPSS version 26.0 (IBM Corp., Armonk, NY). All continuous variables are presented as mean ± standard deviation (SD), or median with interquartile range (IQR), depending on the distribution. Normality was tested with normality plots and the Shapiro-Wilk test, and group comparisons were done with the Student *t* test or the Mann-Whitney *U* test. Categorical variables are presented as frequencies with percentages, and were compared with the χ^2^ test. The correlation of calcification index and calcium score was tested with bivariate correlation and described with the Pearson coefficient. Binary logistic regression analysis was used to identify variables associated with access-site-related vascular or bleeding complications after MANTA closure. Cutoff values for tortuosity index and angulation were selected by visual analysis of their empirical receiver operating characteristic curves (vascular and bleeding complications registered). Both variables were added as categorical variables to the univariable regression model. For the multivariable model, the tortuosity index and angulation were merged as one categorical variable with 3 categories—high TI, high angulation, or both. A *P* value < 0.05 was considered statistically significant.

## Results

### Baseline characteristics

Within the study period, 109 eligible patients consented to participate in our registry. Their baseline characteristics are summarized in Table 1. Most of these elderly patients (median age: 79 years [IQR 75-81 years]) were at intermediate surgical risk (Logistic EuroScore median 7.24 [IQR 4.61-10.84]), had hypertension (88%) and hypercholesterolemia (77%), and used at least one type of anticoagulant or antiplatelet agent (81%). The prevalence of peripheral artery disease was rather low (11%).

**Table 1:** Baseline characteristics

Characteristic	All patients (n = 109)	With complication (n = 43)	Without complication (n = 66)	*P*
Age, y	79 [75–81]	79 [75–81]	79 [74–82]	0.867
Female gender	49 (45)	15 (35)	34 (52)	0.088
BMI, kg/m^2^	27.2 [24.2–30.5]	27.3 [24.3–30.6]	27.2 (24.6–29.8)	0.963
Logistic EuroSCORE	7.24 (4.61–10.84)	6.13 [4.46–9.13]	7.72 [4.73–12.25]	0.223
COPD	16 (15)	6 (14)	10 (15)	0.863
Peripheral artery disease	12 (11)	3 (7)	9 (14)	0.358
Hypercholesterolemia	77 (71)	32 (74)	45 (68)	0.485
Pulmonary hypertension	36 (33)	15 (35)	21 (32)	0.739
Hypertension	96 (88)	37 (86)	59 (89)	0.598
History of CVA or TIA	19 (17)	8 (19)	11 (17)	0.794
Prior cardiac surgery	15 (14)	5 (12)	10 (15)	0.602
Aortic valve	4 (4)	1	3	
Mitral valve	1 (1)	1	0	
Preoperative hemoglobin, mmol/L	8.0 ± 0.96	8.2 ± 0.84	7.9 ± 1.02	0.118
Preoperative kidney function, ml/min per 1.73 m^2^	63 [47–76]	68 [52–76]	61 [47–76]	0.466
Severely decreased kidney function (GFR < 30)	11 (10)	3 (7)	8 (12)	0.522
Aortic valve area, cm^2^	0.8 [0.7–0.9]∗	0.80 [0.66–0.90]	0.80 [0.70–0.96]	0.314
Mean pressure gradient, mm Hg	44 [36–60]†	49 (39–63)	40 [34–53]	0.062
Left ventricular ejection fraction, %	55 [45–60]	55 (45–60)	55 [45–60]	0.660
Type of antiplatelet or anticoagulant therapy				0.962
OAC	15 (14)	7 (16)	8 (12)	
NOAC	24 (22)	9 (21)	15 (23)	
Antiplatelet therapy	47 (43)	18 (42)	29 (44)	
(N)OAC and antiplatelet agent	2 (2)	1 (2)	1 (2)	
INR‡	1.05 [1.01–1.15]	1.06 [1.02–1.17]	1.04 [1.01–1.14]	0.518

Values are n (%), mean (± standard deviation), or median [interquartile range], unless otherwise specified.

BMI, body mass index; COPD, chronic obstructive pulmonary disease; CVA, cerebrovascular accident; EuroSCORE, **Euro**pean **S**ystem for **C**ardiac **O**perative **R**isk **E**valuation; GFR, glomerular filtration rate; INR, international normalized ratio; NOAC, non-vitamin K oral anticoagulant; OAC, oral anticoagulant, vitamin K antagonist; TIA, transient ischemic attack.

∗ Aortic valve area n = 98.

† Mean pressure gradient n = 93.

‡ INR n = 100.

### Access site and valve placement

In almost all patients (97%), a right-sided approach was used, and in 23%, femoral arterial access was obtained under ultrasound guidance. In 8.7%, more than one arterial puncture attempt was required. Interventional cardiologists performed 76% of the procedures. Balloon-expandable valves were implanted in 60% of the patients. Operators closed all TAVI access sites with an 18 Fr-sized MANTA. Patients with vs without TAVI access-site-related vascular and bleeding complications (major and/or minor) had comparable 30-day and 1-year mortality rates (Table 2). Patients with major vascular and bleeding complications had higher 1-year mortality than those without (3 [30%] vs 6 [6.1%]). In 7 cases, the cause of death is unknown; the other deaths were not access-site-related (hemorrhagic stroke and cancer).

**Table 2:** Procedural characteristics and post-procedural survival

Total study sample	All patients (n = 109)	Vascular/bleeding complications (n = 43)	No vascular/bleeding complications (n = 66)	*P*
Echo-guided puncture	25 (23)	14 (33)	11 (17)	0.054
Right-sided access site	106 (97)	43 (100)	63 (95)	0.156
Puncture attempts > 1∗	8 (8.7)	4 (11.8)	4 (6.9)	0.461
Self-expanding valve	44 (40)	22 (51)	22 (33)	0.064
Sheath type∗				0.255
Cook	21 (20.6)	11 (28.9)	10 (15.6)	
eSheath	65 (63.7)	21 (55.3)	44 (68.8)	
Sheathless	16 (15.7)	6 (15.8)	10 (15.6)	
Sheath size, Fr∗	16 [14–16]	16 [14–20]	15 [14–16]	0.107
18-Fr-sized MANTA	109 (100)	43 (100)	66 (100)	—
Flow stop measurement, cm∗	3.5 [3.0–4.0]	3.5 [3.0–4.0]	3.5 [3.0–4.0]	0.436
Surgeon as the first operator	26 (24)	7 (16)	19 (29)	0.134
Total procedure time, min	34 [28–45]	37 [28–49]	34 [26–42]	0.148
Mortality at 30-d follow-up	0	0	0	NA
Mortality at 1-y follow-up	9 (8.3)	4 (9.3)	5 (7.6)	0.737

Values are n (%) or median [interquartile range], unless otherwise indicated.

NA, not applicable.

∗ Missing data; arterial puncture attempts n = 92; sheath type n = 102; sheath size n = 102; MANTA vascular closure device (Teleflex Inc., Wayne, PA) flow-stop measurement n = 92; eSheath = expandable sheath; Cook = Cook Check-Flo Performer-Sheath (Cook Medical, Bloomington, IN).

### Assessment of the iliofemoral anatomy

**Figure 2:**
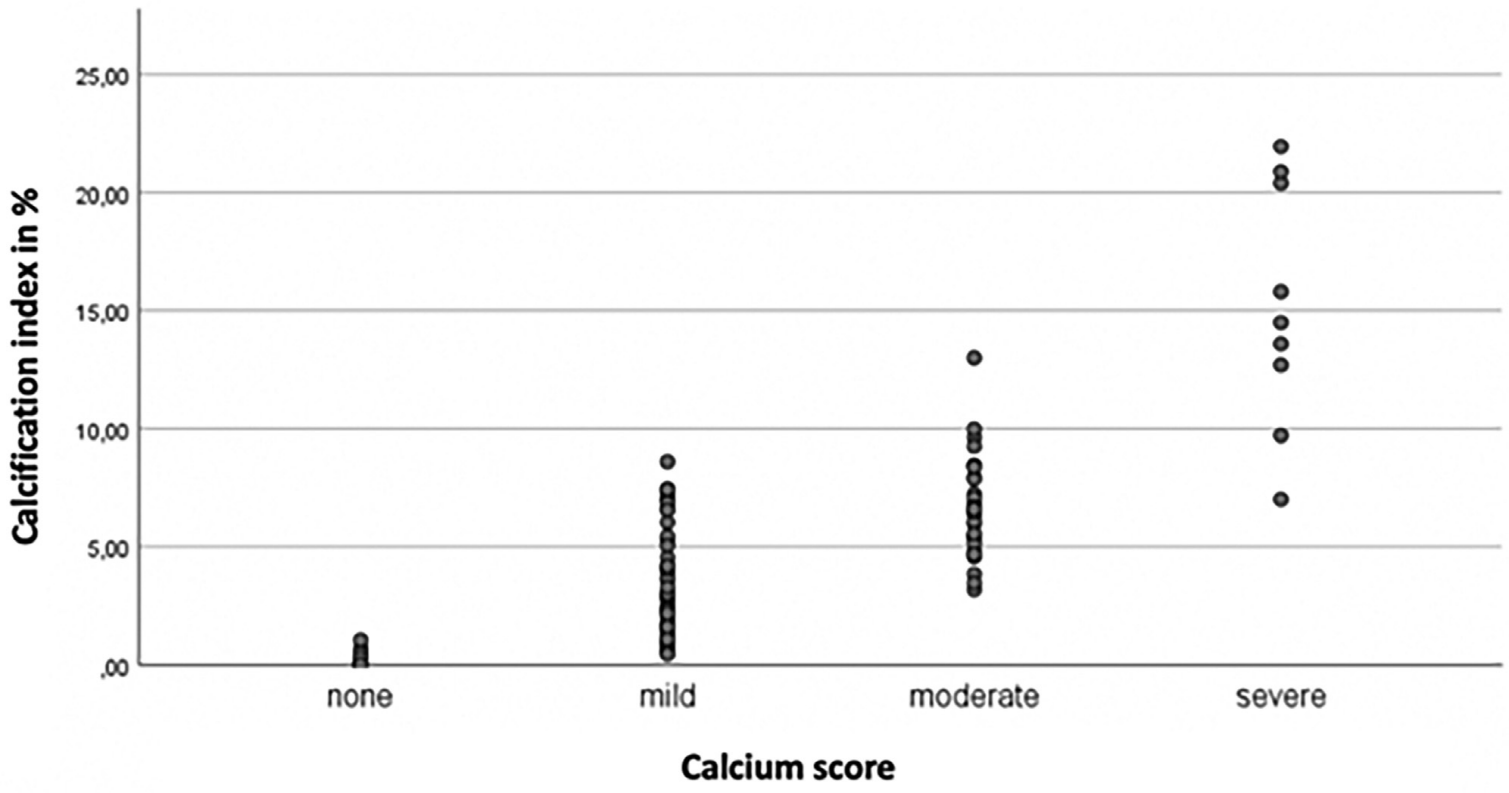
The relationship between the calcium score given by the researcher and the measured calcification index.

Tortuosity index was significantly higher in the group with access-site-related vascular or bleeding complications, compared to the group without (26% [18%-33%] vs 19% [13%-29%], respectively; *P* = 0.012) as well as maximal angulation of the access site (50° [40°-59°] vs 43° [36°-51°], respectively; *P* = 0.026). Calcification index, calcium score, and minimal arterial lumen diameter across the iliofemoral system on the TAVI side were comparable between groups (Table 3). The calcium index did show a good correlation with the widely used semiquantitative calcium score (Pearson correlation: 0.818, *P* < 0.001; Fig. 2).

**Table 3:** Assessment of the transfemoral aortic valve implantation access site

Total study sample	All patients (N = 109)	Complications (n = 43)	No complications (n = 66)	P
Direct distance, mm	183.4 ± 19.5	179.8 ± 20.6	185.7 ± 18.5	0.120
CLF distance, mm	226.5 ± 23.7	226.3 ± 24.2	226.6 ± 23.6	0.948
Tortuosity index,∗ %	22 [15–31]	26 [18–33]	19 [12–29]	0.012
Maximal angulation, °	46 [38–55]	50 [40–59]	43 [36–51]	0.026
Calcium score				0.811
None	9 (8.3)	3 (7.0)	6 (9.1)	—
Mild	62 (56.0)	24 (55.8)	37 56.1)	—
Moderate	30 (27.5)	11 (25.6)	19 (28.8)	—
Severe	9 (8.3)	3 (7.0)	4 (6.1)	—
Ca volume, mm^3^	673 [281–1141]	684 [334–1176]	671 [198–1121]	0.564
Vessel volume, mm^3^	16,577 [13,941–20,412]	16,530 [14,081–22,841]	16,688 [13,727–20,090]	0.480
Calcification index,† %	3.94 (1.62–6.86)	3.47 (2.17–6.60)	4.15 (1.36–6.90)	0.728
MALD, mm	7.0 (6.3–7.3)	7.0 (6.3–7.7)	6.9 (6.0–7.3)	0.139

Data are presented as mean ± standard deviation, median [interquartile range], or n (%), unless otherwise indicated. All measurements were done between the aortic bifurcation and the femoral bifurcation. In this table, only the measurements for the access-site side are reported.

Ca, calcium; MALD, minimal arterial lumen diameter across the iliofemoral system.

∗ Tortuosity index in % = ([centerline of flow / direct distance] – 1) ∗ 100.

† Calcification index in % = (Ca volume / total vessel volume) ∗ 100.

### Primary and secondary clinical outcomes

In total, 43 patients (39%) had one or more primary access-sited-related vascular or bleeding complications. Ten of these patients had a major complication, 8 had a significant drop in their hemoglobin level (> 1.86 mmol/L; VARC-2 cutoff value), and 1 had a perforation at the access site. The remaining 34 patients had minor complications, hematomas or a pseudoaneurysm (Table 4).

**Table 4:** Access-site-related vascular and bleeding complications after MANTA vascular closure device (Teleflex Inc., Wayne, PA) closure

Major bleeding complications	8 (7.3)
Hemoglobin drop > 1.86 mmol/L∗	8
- Pseudoaneurysm + access-site hematoma	1
- Large access-site hematoma	6
- Small access-site hematoma	1
Major vascular complications	2 (1.8)
Perforation	2
- Surgical repair	2
Minor vascular complications	3 (2.8)
Pseudoaneurysm	3
- Surgical repair	3
Minor bleeding complications	31 (28.4)
Access-site hematoma	31
- Pressure bandage	12

Values are n, or n (%). In-hospital complications, N = 109. If one patient had more complications, only the most severe one was scored. For “hemoglobin drop,” the causes are shown; for the others, the applied treatment is shown. Hemoglobin levels were only included up to and including postoperative day 4.

∗ Cutoff value per Valve Academic Research Consortium (VARC)-2.

Direct arterial hemostasis was achieved in 77 patients (70.6%). Additional manual compression for over 10 minutes was necessary for 5 patients (4.6%). Pressure bandage was required in 25 patients (22.9%), and surgical intervention was required in 2 patients (1.8%). Postoperative hospitalization was longer for patients with access-site-related complications ( > 3 days: 40% vs 21%; *P* = 0.020). After discharge, we did not register any major access site-related vascular and bleeding complications.

### Tortuosity and the risk of vascular complications

After visual assessment of their respective receiver operating characteristic curves, a cutoff value of 49.5 degrees was chosen for the maximal angulation (sensitivity 57%; specificity 70%), and 22.8% was chosen for the tortuosity index (sensitivity 62%; specificity 61%). Univariable binary regression analysis showed that only maximal angulation and tortuosity index could have had a significant effect on TAVI access-site related vascular and bleeding complications (major and minor), with odds ratios (ORs) of 3.4 (*P* = 0.005) and 2.8 (*P* = 0.012), respectively. The effect of other, traditional risk factors remained insignificant (Supplemental Table S1) We added the combination of these variables (high tortuosity index, high degree of angulation, or both), together with an echo-guided puncture, to an age- and gender-adjusted multivariable model. Patients with both high angulation and significant tortuosity had an increased risk for access-site-related vascular complications (OR 4.7, confidence interval [1.65-13.20]; *P* = 0.004; Supplemental Table S2) Although the event rate of major complications was relatively low, the same angulation cutoff proved to be a predictor of major complications as well (*P* = 0.017; OR 7, confidence interval [1.4-34.8]).

## Discussion

This prospective single-centre study identified arterial tortuosity, elongation, and especially angulation of the iliofemoral arteries as important predictors of access-site-related vascular and bleeding complications in TF TAVI procedures using the MANTA closure device. Also, our results indicate that routine, semi-objective screening of preoperative computed tomography scans cannot prevent all tortuosity-related bleeding complications.

The incidence of major vascular and bleeding complications and unplanned vascular repairs has decreased within the intermediate- and lower-risk population, compared to that in the first high-risk TAVI trials. Yet, both our data and the available literature show that these events still affect short- and long-term outcomes in up to 4%-7.5% of the TAVI population.^1^^,^^6^^,^^7^^(p3),^^10^^,^^20^ The incidence of major vascular and bleeding events in our cohort matches the aforementioned historical data, although we observed more minor complications than most other groups reporting on minor events (∼3%-30%).^8^, ^9^, ^10^^,^^21^ A plausible explanation for this difference could be our close and prospective monitoring of events and the slight differences in definitions.

Proper preprocedural planning, taking patient- and device-related factors into account, is crucial to minimalize the incidence of periprocedural bleeding and vascular complications. The interaction of anatomy and closure devices is especially interesting, as these devices have unique learning curves, usability, and applicability. To these ends, several predictors of adverse events have been proposed.^10^^,^^12^ Circumferential calcification^22^ and sheath-to-femoral artery ratio^8^^,^^22^ are widely known predictors, and recent publications suggest that off-target puncture, unfavourable arteriotomy phenotype, small artery diameter, left femoral access, and female gender all increased the risk of vascular-closure-device-related vascular complications.^10^^,^^11^ In our cohort, left femoral access use was negligible, and data on circumferential calcification were not available. We did determine a normalized iliofemoral calcification index, but this showed no relationship with the registered complications. Our preprocedural screening takes circumferential calcifications and arterial diameters, and the artery-sheath ratio, into account, and this selection bias probably diminished their effect on our study population.

Our most important and novel finding is the unfavourable influence of arterial elongation, measured by the tortuosity index and angulation, on the efficacy of a plug-based vascular closure device (MANTA). We have shown that a tortuosity index of 22.8 and an angle > 49.5 are important and synergistic predictors of VARC-2 vascular and bleeding complications. These findings connect well to the findings of Mach et al., who published similar findings (a tortuosity score of 21.2) in a population treated with suture-based closure devices and clinical experience with endovascular aneurysm repair.^13^^,^^15^^,^^16^ A possible explanation could be the distortion of tortuous vessels, caused by the guidewire and temporary placement of the intravascular parts of the closure devices. This possibility may explain our findings of local bleeding complications and the need for additional manipulations to achieve complete hemostasis (30% of our patients).

We believe that objective tortuosity measurements should be incorporated into the preprocedural screening process to improve procedural outcomes. Both measurements could help with closure-device selection and in predicting access-site-related vascular and bleeding complications, although further studies are warranted to determine and validate the most relevant cutoff values.

### Strengths and limitations

The strength of the study was its prospective design, with predefined access-site evaluation immediately after and during hospitalization. Patients were treated with one type of closure device. Access-site assessment was scored by the ward doctors. We faced a limited follow-up after hospital discharge due to pandemic measurements (COVID-19)—from March 2020 onward, patients could not be physically present in the outpatient clinic for the 6-week follow-up appointment and instead received a telephone consultation. Some of the vascular complications (eg, pseudoaneurysms) may have been missed, as no routine echocardiographic control of the access site was performed. No information is available on circumferential calcifications. Due to the small sample size, information on the influence of complications or other procedural factors on patient survival remains limited.

## Conclusion

Steep angles and greater elongation, as measured by the tortuosity index of the iliofemoral tract, increase the risk of vascular and bleeding complications after utilization of plug-based closure devices in TAVI patients. Integrating these measurements into the TAVI workup could help improve TAVI outcomes.
