# Supplementary material for: Iliofemoral Tortuosity Increases the Risk of Access-Site-Related Complications After Aortic Valve Implantation and Plug-Based Access-Site Closure
Source: CJC Open. 2022 Mar 19;4(7):609–16. doi: 10.1016/j.cjco.2022.03.006 (PMC9294987; doi:10.1016/j.cjco.2022.03.006)
Supplement: Supplementary Tables S1 and S2 [file mmc1.pdf]

SUPPLEMENTARY MATERIAL

Supplemental Table S1. *Univariable binary logistic analysis of various potential predictors of vascular and bleeding complications.*

	OR	CI	P
Female gender	0.504	0.229-1.113	0.09
Semi-quantitative calcium score			
- Mild	1.297	0.296-5.688	0.730
- Moderate	1.158	0.240-5.578	0.855
- Severe	2.5	0.370-16.888	0.347
Calcium volume (mm3)	1.000	1.000-1.000	0.816
Echo guided puncture	2.414	0.973-5.989	0.057
Left-sided puncture	0.000	0.000-	0.999
Number of punctions	1.800	0.420-7.720	0.429
Minimal iliofemoral diameter	0.920	0.752-1.126	0.419
Sheath-to-artery ratio (iliofemoral min. diameter)	0.592	0.119-2.993	0.521
Angulation >49.5	3.436	1.533-7.699	0.003
Tortuosity index >22.8	2.767	1.252-6.120	0.012
Combination score			
- >49.5° or >22.8	2.722	1.011-7.332	0.048
- >49.5° and >22.8	5.115	1.890-13.847	0.001

Supplemental Table S2. *Age and gender corrected multivariable analysis of the predictors of vascular and bleeding complications*

	OR	CI	P
Age (years)	0.970	0.894-1.052	0.463
Female gender	0.504	0.229-1.113	0.09
Echo guided puncture	1.952	0.745-5.114	0.173
Combination score			
- >49.5° or >22.8	2.722	1.011-7.332	0.048
- >49.5° and >22.8	5.115	1.890-13.847	0.001
